# PGC-1α Promotes Ureagenesis in Mouse Periportal Hepatocytes through SIRT3 and SIRT5 in Response to Glucagon

**DOI:** 10.1038/srep24156

**Published:** 2016-04-07

**Authors:** Lulu Li, Ping Zhang, Zhengxi Bao, Tongxin Wang, Shuang Liu, Feiruo Huang

**Affiliations:** 1 Department of Animal Nutrition and Feed Science, College of Animal Science and Technology, Huazhong Agricultural University, Wuhan 430070, China

## Abstract

Excess ammonia is produced during fasting when amino acids are used for glucogenesis. Together with ureagenesis, glucogenesis occurs in periportal hepatocytes mediated mainly through the peroxisome proliferator-activated receptor gamma coactivator 1-alpha (PGC-1α). *In vivo* experiments showed that fasting strongly stimulated mice glucagon secretion, hepatic PGC-1α, sirtuin 3 (SIRT3) and sirtuin 5 (SIRT5) expression and ureagenesis enzymatic activity such as carbamoyl phosphate synthetase 1 (CPS1) and ornithine transcarbamoylase (OTC). Interestingly, ^15^N-labeled urea and ^13^C-labeled glucose production in wild-type mice were significantly increased compared with PGC-1α null mice by [^15^N,^13^C]alanine perfused liver. Glucagon significantly stimulated ureagenesis, expression of SIRT3, SIRT5 and the activities of CPS1 and OCT but did not stimulate PGC-1α silencing hepatocytes in mice periportal hepatocytes. Contrarily, PGC-1α overexpression significantly increased the expression of SIRT3, SIRT5 and the activities of CPS1 and OTC, but induced no significant changes in CPS1 and OTC expression. Morever, SIRT3 directly deacetylates and upregulates the activity of OTC, while SIRT5 deacetylates and stimulates the activity of CPS1. During fasting, PGC-1α facilitates ureagenesis in mouse periportal hepatocytes by deacetylating CPS1 and OTC modulated by mitochondrial deacetylase, SIRT3 and SIRT5. This mechanism may be relevant to ammonia detoxification and metabolic homeostasis in liver during fasting.

During fasting, the nutrient and hormone-dependent regulation plays a vitial role in promoting energy balance and metabolic homeostasis by stimulating glucose output, lipid breakdown and catabolism of aminoacids (AA) in the liver^1^,^2^,^3^. During prolonged fasting, a time when AA becomes an important energy source, the flow of carbon from AA into central metabolism is promoted by hepatic gluconeogenesis^3^,^4^,^5^. Under these conditions there is excess ammonia production, which is converted to urea for ammonia detoxification^6^,^7^. Understanding the central role of hepatic ureagenesis is critical as dysregulated ureagenesis and ammonia levels are often linked to hyperammonemia and hepatic encephalopathy^8^,^9^. Ureagenesis is under tight nutritional and hormonal control^10^. Glucagon is well konwn as a regulator mainly secreted during fasting to maintain metabolic homeostasis^11^. However, the complex regulatory network involving hormone and transcriptional coactivators in urea cycle is still unclear.

Hormonal and nutritional regulation of hepatic gluconeogenesis occurs mainly through modulation of the transcriptional coactivator peroxisome proliferator-activated receptor gamma coactivator 1-alpha (PGC-1α)^12^. In terms of its contribution to the problem of diet-dependent maintenance of energy homeostasis, there is a body of evidence to suggest that PGC-1α is important for the compensatory metabolic responses that occur during food deprivation^13^. In fasting, glucagon signals increase hepatic glucose output through the mediated of PGC-1α^2^,^12^,^13^. Sirtuins have been show to play a role as metabolic sensors that also respond to changes in the energy status and in modulating the activities of key metabolic enzymes^14^. Sirtuins are a family of NAD-dependent protein deacetylases^15^. In mammals, there are seven sirtuins (SIRT1-7), three of which (SIRT3-5) are located in the mitochondrial matrix^3^,^14^. These regulators are increasingly recognized as an important post-translational modification for a number of key metabolic pathways, such as the urea cycle. During fasting SIRT3 and SIRT5 are reported to promote ureagenesis^16^,^17^,^18^. Interestingly, new evidences suggest that SIRT3 and SIRT5 do interact with PGC-1α in liver^15^,^19^.

Highly specialized hepatocytes efficiently maintain the numerous metabolic functions of the liver^20^. Based on the location of the blood vessels and the direction of the blood flow, hepatocytes can be divided into upstream or periportal and downstream or perivenous^21^. Notabaly, hepatocytes show remarkable differences in the levels and activities of various metabolic enzymes according to their periportal or perivenous location^21^. Interestingly, the key enzymes of gluconeogenesis and ureagenesis are preferentially expressed in periportal area^20^,^22^,^23^,^24^,^25^. However, whether glucogenesis promoter PGC-1α is also responsible for ammonia detoxification to compensante the increase of aminoacid catabolism in fasting.

In the present study PGC-1α null mice and mice periportal hepatocytes (control, PGC-1α knockdown and overexpression) and ^15^N-ammonium chloride or [^15^N,^13^C]alanine were utilized as metabolic tracers and nitrogen sources for ureagenesis in perfused liver and periportal hepatocytes. Also, the regulatory mechanism of ureagenesis during fasting and administration of glucagon was demonstrated.

## Methods

### Animal preparation and treatment

The animal handling protocol followed in this study was approved by the Animal Care and Use Committee of College of Animal Sciences and Technology, Huazhong Agricultural University, and was in compliance with the National Research Council’s Guide for the Care and Use of Laboratory Animals. Male 8–12 weeks wild-type C57BL/6 littermates obtained from the Model Animal Research Center of Nanjing University, China, were used for this study. The PGC-1α null mice were obtained from Jackson Laboratory^26^. The mice were housed in a controlled environment with 12 h light/dark cycles and fed a standard rodent chow. The twelve wild-type mice were equally divided into fed and 48 h-fasting groups (n = 6 per group). Tail blood samples were collected to measure the glucagon and glucose concentrations at 0, 12, 24, 36 and 48 h after food deprivation. The fed mice were killed after the first blood collection, while the fasting animals were killed until the last blood collection. Liver samples were immediately frozen in liquid nitrogen and stored at −70 °C until analyzed.

### Liver perfusion

After an overnight (12 h) food-deprived, the livers from wild- and PGC-1α null mice (weighing about 15 ~ 20 g) were perfused in non-recirculating mode as described by Sies^27^ and Brosnan *et al*.^28^. The basic perfusion medium was a Krebs’ saline containing 2.1 mmol lactate, 0.3 mmol pyruvate as metabolic fuels continuously gassed with 95% O_2_ and 5% CO_2_. The perfusion flow rate, pH, pCO_2_, and pO_2_ (in influent and effluent media) were monitored throughout, and the oxygen consumption was calculated. After 20 min of perfusion, either 1 mM [^15^N, ^13^C]alanine (99% enriched in ^15^N and ^13^C; Cambridge Isotope Laboratories) or 0.3 mM ^15^NH_4_Cl (99% enriched in ^15^N; Cambridge Isotope Laboratories), were added to the medium. Perfusions continued for a total of 80 min. Control perfusions consisted of a saline infusion for 20–80 min. Samples were taken at 0, 20, 40, 60 and 80 min from both the influent and effluent media for chemical and GC-MS analysis. At the end of the perfusion, the livers were freeze-clamped with aluminum tongs precooled in liquid N_2_ for urea and glucose assays.

### Isolation and identification of hepatocytes

Mice periportal and perivenous hepatocytes were isolated and enriched by combined digitonin-collagenase perfusion of the liver following the procedure of Taniai *et al*.^29^ and Braeuning *et al*.^21^. The liver was perfused for 10 min with Krebs⁄Henseleit buffer. To obtain periportal hepatocytes, a 5 mM digitonin solution was infused for 10 s through the vena cava and then immediately flushed out from the opposite direction. To obtain perivenous hepatocytes, the digitonin solution was infused through the portal vein. Then, the liver was perfused with collagenase solution, and cells were separated by density gradient centrifugation. Cell viability (assessed by Trypan blue exclusion) was always greater than 85%. The efficiency of separation of periportal and perivenous hepatocytes was determined by western blotting for marker proteins.

### PGC-1α silencing and PGC-1α overexpression

Primary cultured periportal hepatocytes were individually transfected with PGC-1α, SIRT3 and SIRT5 siRNAs or scrambled control siRNA to silence target proteins. The hepatocytes were transfected with Lipofectamine 2000 (Invitrogen) using 150 pmol/well of the following siRNAs: SIRT3 siRNA (Production no. sc-61556, Santa Cruz); SIRT5 siRNA (Production no. sc-63027, Santa Cruz); PGC-1α siRNA (Production no. sc-38885, Santa Cruz) and scrambled (SCR, as control, Sigma-Aldrich) according to the manufacturer’s instructions. The siPGC-1α, siSIRT3 and siSIRT5 samples and the corresponding controls were detected after 24 h. Alternatively, to obtain PGC-1α overexpression hepatocytes, cells were cultured in Williams’ E medium (SigmaAldrich), containing either PGC-1α-expressing adenoviruses (PGC-1α-Ad) or LacZ-Ad (control) at MOI 0.1 according to Buler *et al*.^19^. Preparation of the Ad-PGC-1α virus has been described previously^30^. The cells were detected 48 h after infection.

### Periportal hepatocytes culture and treatment

For the starvation experiments the medium was replaced by HBSS supplemented with 10 mM HEPES and an antibiotic solution (Amresco) as previously described^3^. Periportal hepatocytes transfected with corresponding siRNAs were incubated with 4 mM L-alanine at 37 °C for 4 h in the presence or absence (control) of 1 μM glucagon. All the experiments were done in the presence of L-ornithine (4 mM, Sigma) and contained pyruvate as a source of aspartate to provide additional nitrogen in urea^31^,^32^. At the end of the experiments, hepatocytes were harvested for urea, mRNA and proteins determination.

### Preparation of mitochondrial and submitochondrial fractions

Mitochondria were isolated from the livers of fed and 24 hr-fasted mice as well as various treated hepatocytes as described by Shimizu *et al*.^33^ and Nakagawa *et al*.^3^. Firstly, the livers and hepatocytes were homogenized with a glass-Teflon Potter homogenizer. Mitochondria were isolated in medium containing 0.3 mmol mannitol, 10 mmol potassium Hepes (pH7.4) and 0.2 mmol EGTA (pH7.4) and then washed twice and suspended in the same medium without EGTA. Mitochondrial subfractionation was performed as previously described^3^,^34^ to obtain mitochondrial membrane and matrix fractions.

### RNA isolation and quantitative real-time PCR

Total RNA was extracted from the livers and hepatocytes using TRIzol reagent (Invitrogen Corporation, Carlsbad, CA, US) according to the manufacturer’s specifications. Reverse transcription-PCR and real-time quantitative PCR analysis were performed. Briefly, reverse transcription of total RNA was performed using an avian myeloblastosis virus RT with a first-strand complementary DNA synthesis kit for reverse transcription-PCR. Aliquots of the reverse transcription reactions were then submitted in duplicate to online quantitative PCR with the LightCycler^®^ 480 Real-Time PCR system (Roche Applied Science) with SYBR green using the FastStart DNA-Master SYBR Green I kit (Roche Applied Science). Relative abundance of mRNA was calculated after normalization to 18S ribosomal RNA. Specific primers were synthesised commercially (Shanghai Sangon Biological Engineering Technology and Services Company Ltd, Shanghai, China). Sequences for the primers used in this study are shown in Table S1.

### Western blotting and immunoprecipitation

Marker proteins in hepatocytes were determined by western blotting, as recently described^21^,^35^ using antibodies against glutamine synthetase (1:5000 dilution, Sigma), E-cadherin (1:1000 dilution, Santa Cruz), G-protein-coupled receptor 49 (1:1000 dilution, Abcam), glyceraldehyde-3-phosphate dehydrogenase (1:1000 dilution, Abcam) and cytochrome P450 1A (1:500 dilution, Abcam). After treatment nuclear extracts prepared from livers and hepatocytes were analyzed by immunoblotting using PGC-1α antibody (1:1000 dilution, Abcam) to detect PGC-1α protein. In addition, the cytosolic and mitochondrial fractions were also isolated and subjected to Western blot analysis. Proteins were detected using antibodies against phosphoenolpyruvate carboxykinase (PEPCK), glucose-6-phosphatase (G6Pase), α-Tubulin, SIRT3, SIRT5, CPS1, OTC and mtHSP70 (1:1000 dilution, Abcam). For immunoprecipitation, livers and hepatocytes mitochondrial lysates were performed with anti-OTC or anti-CPS1 antibody overnight at 4 °C, then added with protein A/G beads for 4 h followed by western blotting using anti-acetyl-lysine antibody (1:1000 dilution, Abcam).

### Enzymatic activity assay

A colorimetric method was used to measure converted citrulline to evaluate the activities of CPS1 and OTC^3^,^36^. For determination of the CPS1 activity, mitochondria matrix lysates were incubated in CPS1 assay buffer. The supernatant was separated by centrifugation and used for color development. For each assay, 100 μl supernatant and 600 μl color development solution were mixed, boiled at 100 °C for 15 min and then cooled. The absorbance and its relationship to citrulin concentration were obtained. Then, the unit activity was calculated as follows: unit/mg protein = OD × 0.8 × 3.5 × 6/37.8 × mg protein. For OTC activity assay, the mitochondria matrix lysates were incubated in OTC assay buffer. Subsequent steps were the same as in the CPS1 activity assay.

### Analysis of glucagon, urea and glucose concentrations

The plasma glucose and glucagon concentrations were measured using an automatic biochemical analyzer (Siemens) and a glucagon commercial kit (Linco), respectively. In the perfused experiment, ^15^N-labeled urea-N and ^13^C-labeled glucose from plasm and tissue samples were detected by GC-MS as described by Brosnan *et al*.^28^,^32^. After the hepatocytes were cultivated, the culture medium was aspirated and centrifuged at 500 g for 5 minutes to obtain a cell-free supernatant for determination of urea via the urease method^37^.

### Measurement of ATP levels

Hepatocyte ATP levels were detected using a luciferin- and luciferase-based assay. After transfection of siControl, siPGC-1α for 24 h, respectively, the hepatocytes were washed with PBS and the ATP levels were measured using an ATP determination kit (Invitrogen) according to the manufacturer’s directions.

### Cellular viability and MTT assay

The activity of lactate dehydrogenase (LDH) was used as a measure of cell viability. LDH leakage into the culture media was measured using a LDH assay kit (Biovision) after hepatocytes isolation, 24 h siRNA transfection as well as ammonium chloride, alanine treatment, according to the manufacturer’s instructions. The assay for MTT was carried out as described by Soria *et al*.^8^.

### Statistical analysis

Data are presented as means with their standard errors. Significance was determined using Student’s *t-*test or one-way analysis of variance (Tukey’s test). The level of significance was set at *p* < 0.05. Significant differences are indicated by single asterisk (*) when *p* < 0.05 and double asterisk (**) when *p* < 0.01.

## Results

### Fasting-induced ureagenesis and gluconeogenesis in mice liver

**Figure 1:**
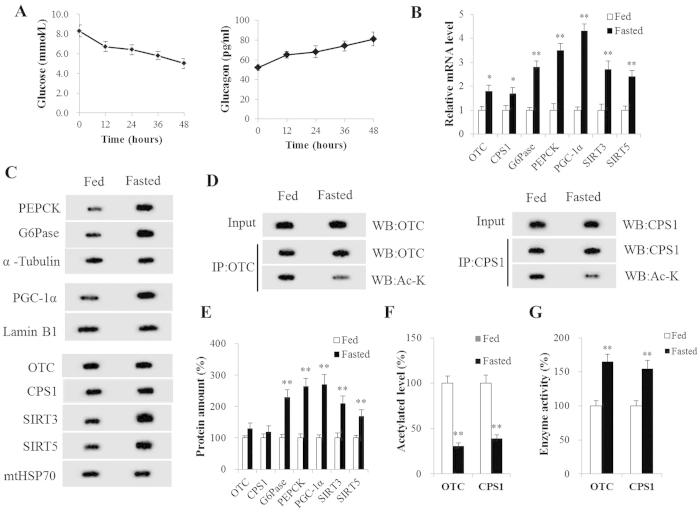
Fasting Induced Ureagenesis and Gluconeogenesis in Mice Liver. (**A**) Blood glucagon (pg/ml) and glucose concentrations (μmol/l) at every 12 h time point. (**B**) OTC, CPS1, G6Pase, PEPCK, PGC-1α, SIRT3 and SIRT5 mRNA levels in mice liver. (**C**,**E**) OTC, CPS1, SIRT3, SIRT5, G6Pase, PEPCK and PGC-1α protein levels in mice liver. (**D**,**F**) Acetylated OTC and CPS1 in mice liver. (**G**) Activity of OTC and CPS1 in mice liver. Liver tissue samples were collected from fed and 48 h fasted mice. Data are expressed as the mean values and standard errors of each different experiment (n = 6 per group). *P < 0.05 from fed group, **P < 0.01 from fed group.

Fasting caused a significant decrease in blood glucose concentration throughout the experiment, while blood glucagon increased and peaked after a 48 hr-fast (Fig. 1A). A sharp increase in glucagon and decrease in blood glucose levels was observed at 12 h after fasting (Fig. 1A). In the present study, the fasting samples of liver tissue were collected from 48 h fasted mice. The first and last step in liver gluconeogenesis was catalyzed by PEPCK and G-6-Pase, respectively. We found that fasting induced a markedly higher expression (*P* < 0.01) of hepatic PEPCK and G-6-Pase both in mRNA and protein levels (Fig. 1B,C,E). Mitochondrial CPS1 and OTC are the first and second catalytic enzyme steps of the urea cycle (CPS1 as the rate-controlling enzyme) and were significantly more abundant in mRNA in liver of fasted than in fed mice (P < 0.05, Fig. 1B). Still, the protein expression of CPS1 and OTC did not differ (*P* > 0.05) between the two groups (Fig. 1C,E). Interestingly, compared to fed mice, the acetylation levels of CPS1 and OTC were significant (*P* < 0.01) lower, while the activities of CPS1 and OTC were significantly (*P* < 0.01) higher in fasted mice liver (Fig. 1D,F,G). Notably, fasting also strongly stimulated (*P* < 0.01) PGC-1α, SIRT3 and SIRT5 expression in mice livers (Fig. 1B,C,E).

### Ureagenesis and gluconeogenesis in perfused livers

**Figure 2:**
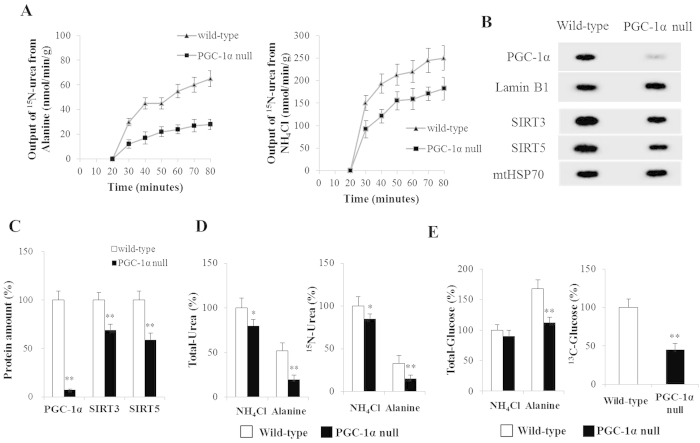
Ureagenesis and Gluconeogenesis in Perfused Livers. (**A**) ^15^N-labeled urea (urea isotopomer containing one (U_m + 1_) ^15^N) output. (**B**,**C**) PGC-1α, SIRT3 and SIRT5 protein levels in wild- and PGC-1α null mice liver. (**D**) Percent ^15^N-labeled and total urea in wild-type liver perfused with ammonium chloride. (**E**) Percent ^13^C-labeled glucose in wild-type liver perfused with alanine. Percent total glucose in wild-type liver perfused with ammonium chloride. Data are expressed as means and standard errors of each experiment (n = 4 per group). *P < 0.05 from fed group, **P < 0.01 from fed group.

Ammonium chloride and alanine were used as nitrogen sources (alanine also as carbon source) to investigate hepatic ureagenesis and gluconeogenesis in wild- and PGC-1α null mice. Figure 2A shows the changes of ^15^N-labeled urea in the effluent under various experimental conditions. During perfusion (20 ~ 80 min), Wild-type mice livers perfused with either alanine or ammonium chloride had a higher ^15^N-labeled urea output than those of PGC-1α null mice. Almost no PGC-1α expression was detected in PGC-1α null mice liver (Fig. 2B,C). The PGC-1α null mice liver showed a lower SIRT3 and SIRT5 protein expression (*P* < 0.01) compared with the liver in wild-type mice (Fig. 2B,C). Consistent with the results in effluent media, the amount of ^15^N-labeled and total urea in wild-type livers tissue samples were markedly larger (*P* < 0.01) than that in PGC-1α null livers with perfusions of alanine (Fig. 2D). However, when perfused with ammonium chloride, PGC-1α null slightly lower ^15^N-labeled and total urea production (Fig. 2D). In addition, compared to ammonium chloride, alanine strongly induced (*P* < 0.01) hepatic gluconeogenesis in wild-type mice (Fig. 2E). Notably, PGC-1α null liver failed to increase (*P* < 0.01) ^13^C-labeled and total glucose production from alanine (Fig. 2E).

### Expression profiles of perivenous and periportal hepatocytes

**Figure 3:**
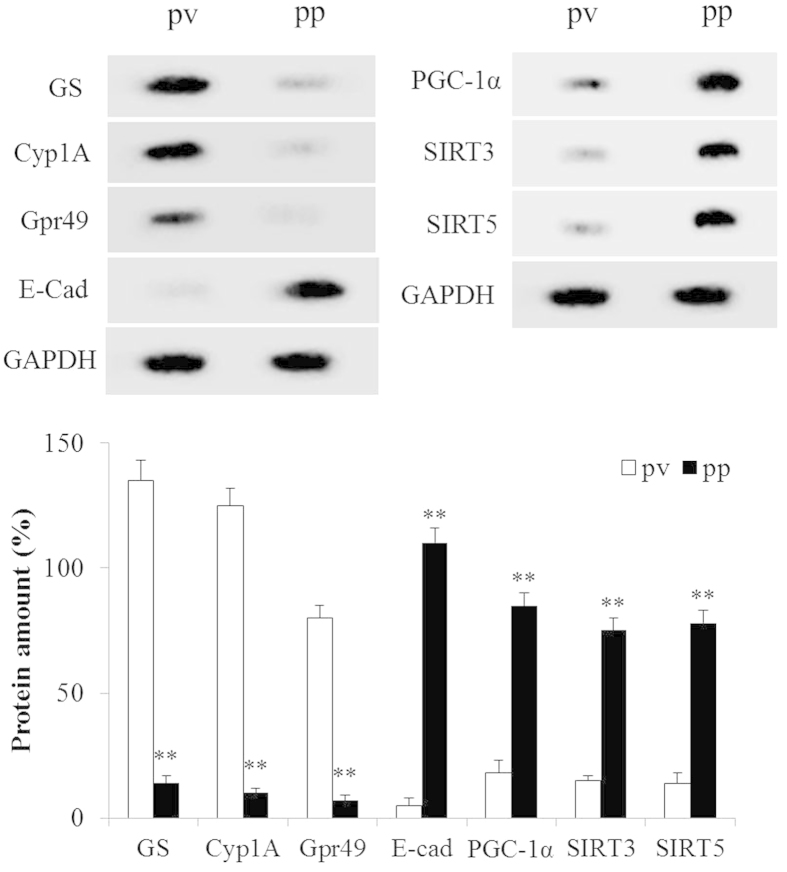
Expression Profiles of Perivenous and Periportal Hepatocytes. Western blot analysis of marker protein levels from perivenous (pv) and periportal (pp) hepatocytes. The indicated proteins specifically expressed in hepatic different zone and chosen as ‘markers’ for periportal and perivenous hepatocytes. GS, glutamine synthetase; E-cad, E-cadherin; Gpr49, G-protein-coupled receptor 49; Cyp1A, cytochrome P450 1A. Morever, PGC-1α, SIRT3 and SIRT5 protein were detected from perivenous and periportal hepatocytes. Glyceraldehyde-3-phosphate dehydrogenase (GAPDH) was used as a loading control. n = 4 for each independent experiments.

Periportal and perivenous hepatocytes were obtained by combined digitonin⁄collagenase perfusion of liver. These hepatocytes have remarkable differences in their levels of protein expression, which can be regarder as markers to distinguish one or the other. The efficiency of hepatocytes separation was detected by western blot and the results are in good agreement with previous reports^21^: E-cadherin was expressed in mice periportal hepatocytes, while glutamine synthetase, G-protein-coupled receptor and cytochrome P450 1A were expressed only in perivenous hepatocytes (Fig. 3). Furthermore, the expression of PGC-1α, mitochondrial SIRT3 and SIRT5 were significantly higher (*P* < 0.01) in periportal hepatocytes than that in perivenous hepatocytes.

### Ureagenesis in PGC-1α knockdown periportal hepatocytes in response to glucagon

**Figure 4:**
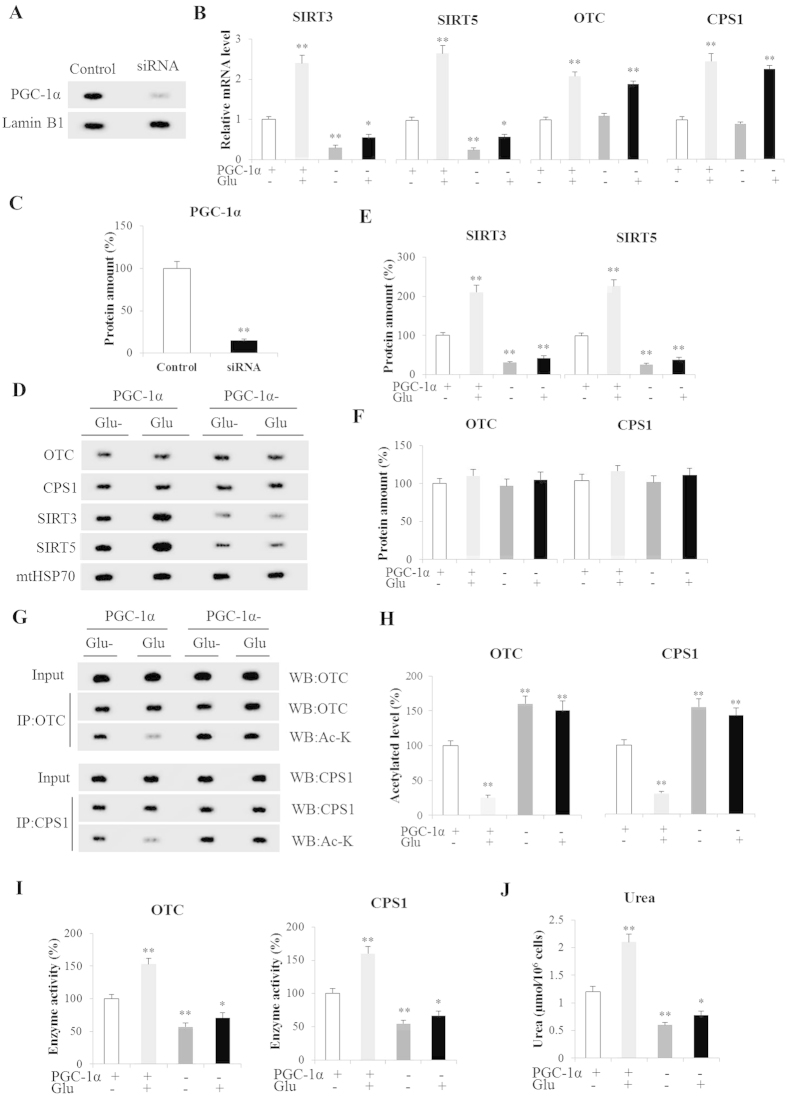
Ureagenesis in PGC-1α Knockdown Hepatocytes in Response to Glucagon. (**A**,**C**) PGC-1α expression in periportal hepatocytes; (**B**) OTC, CPS1, SIRT3 and SIRT5 mRNA levels in control and PGC-1α knockdown periportal hepatocytes; (**D**–**F**) OTC, CPS1, SIRT3 and SIRT5 protein levels were detected in periportal hepatocytes; (**G**,**H**) Acetylated OTC and CPS1 in periportal hepatocytes; (**I**) Activity of OTC and CPS1 in periportal hepatocytes. (**J**) Urea in hepatocytes cultured media. PGC-1α(+) Glu(−) were used as controls. Glu(−) and Glu(+) represent with and without glucagon in medium, respectively. PGC-1α(−) and PGC-1α(+) represent hepatocytes transfected with siPGC-1α and control siRNA, respectively. Data are expressed as the means and standard errors for each different experiment (n = 4 per group). *P < 0.05 from fed group, **P < 0.01 from fed group.

To induce a decrease in PGC-1α expression, primary periportal hepatocytes were transfected with siRNAs containing sequences specific for mice PGC-1α. After transfection, PGC-1α protein expression decreased by approximately 85% (Fig. 4A,C). The effect of PGC-1α knockdown on ureagenesis in response to glucagon is shown in Fig. 4. Glucagon strongly stimulated (*P* < 0.01) ureagenesis in hepatocytes transfected with siRNA SCR but ureagenesis was significantly decreased in PGC-1α-silent hepatocytes both in the presence (*P* < 0.05) and absence (*P* < 0.01) of glucagon (Fig. 4J). Glucagon could also significantly increase SIRT3, SIRT5, OTC and CPS1 mRNA levels by about 2.4-, 2.7-, 2.1- and 2.5-fold, respectively, in hepatocytes transfected with siRNA SCR (*P* < 0.01, Fig. 4B). Likewise, the glucagon-inducible protein expressions of SIRT3, SIRT5, OTC and CPS1 in control hepatocytes were 210% (P < 0.01), 230% (*P* < 0.01), 110% (*P* > 0.05) and 112% (*P* > 0.05), respectively, of those without glucagon treatment (Fig. 4D–F). Notably, the protein expression of SIRT3 and SIRT5 were significantly decreased (*P* < 0.01) in lacking PGC-1α hepatocytes both in the presence and absence of glucagon (Fig. 4D,E). However, no significant changes (*P* > 0.05) in CPS1 and OTC protein expression were observed in PGC-1α-deficient hepatocytes with glucagon administration, even though it could increase their mRNA levels (*P* < 0.01, Fig. 4B,D,F). In addition, glucagon also significantly decreased acetylated CPS1 and OTC levels and increased their activities in control hepatocytes (*P* < 0.01, Fig. 4G,H). Interestingly, the higher activities of CPS1 and OTC were always accompanied to lower acetylation levels. It is worth noting that PGC-1α knockdown markedly upregulated acetylated CPS1 and OTC levels (*P* < 0.01) and downregulated their activities in the presence (*P* < 0.05) and absence (*P* < 0.01) of glucagon (Fig. 4G–I). Thus, PGC-1α silencing strongly reduced (*P* < 0.01) glucagon-induced ureagenesis (Fig. 4J).

### Ureagenesis in PGC-1α overexpression periportal hepatocytes

**Figure 5:**
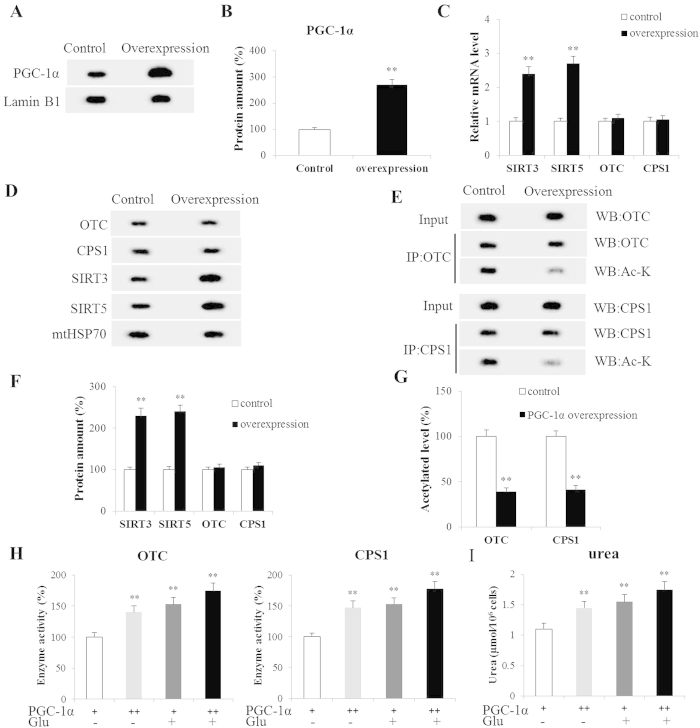
Ureagenesis in PGC-1α Overexpression Hepatocytes. (**A**,**B**) Expression of PGC-1α in periportal hepatocytes; (**C**) Levels of OTC, CPS1, SIRT3 and SIRT5 mRNA in control and PGC-1α overexpression hepatocytes; (**D**,**F**) Protein expressions of mitochondrial OTC, CPS1, SIRT3 and SIRT5 as analyzed by western blot; (**E**,**G**) Acetylated OTC and CPS1 in hepatocytes; (**H**) Activities of OTC and CPS1 in hepatocytes; (**I**) Urea in hepatocytes cultured media. Glu(−) and Glu(+) represent with- and without glucagon in medium, respectively. PGC-1α(+) and PGC-1α(++) represent control and PGC-1α overexpression hepatocytes, respectively. Data are expressed as means and standard errors for each different experiment (n = 4 for each group). *P < 0.05 from control group, **P < 0.01 from control group.

Primary periportal hepatocytes were grown with PGC-1α-expressing adenoviruses (PGC-1α-Ad) to induce an increase in PGC-1α expression. The PGC-1α protein expression was increased 2.7-fold (*P* < 0.01) compared to control (Fig. 5A,B). Notably, the SIRT3 and SIRT5 mRNA levels were induced (*P* < 0.01) by PGC-1α overexpression (Fig. 5C). Likewise, similar trends were observedin SIRT3 and SIRT5 protein expression (*P* < 0.01, Fig. 5D,F). However, the protein expression of CPS1 and OTC was not affected in PGC-1α overexpression hepatocytes (*P* > 0.05, Fig. 5D,F). The acetylated CPS1 and OTC levels were significantly lower while their activities were significantly higher with PGC-1α overexpression (*P* < 0.01, Fig. 5E,G,H). Correspondingly, the PGC-1α overexpression-inducible CPS1 and OTC activities markedly increased ureagenesis (*P* < 0.01, Fig. 5H,I). It is interesting that the addition of glucagon enhanced the effect of PGC-1α on CPS1 and OTC activities and ureagenesis (Fig. 5H,I).

### Effect of SIRT3 and SIRT5 on ureagenesis in periportal hepatocytes

**Figure 6:**
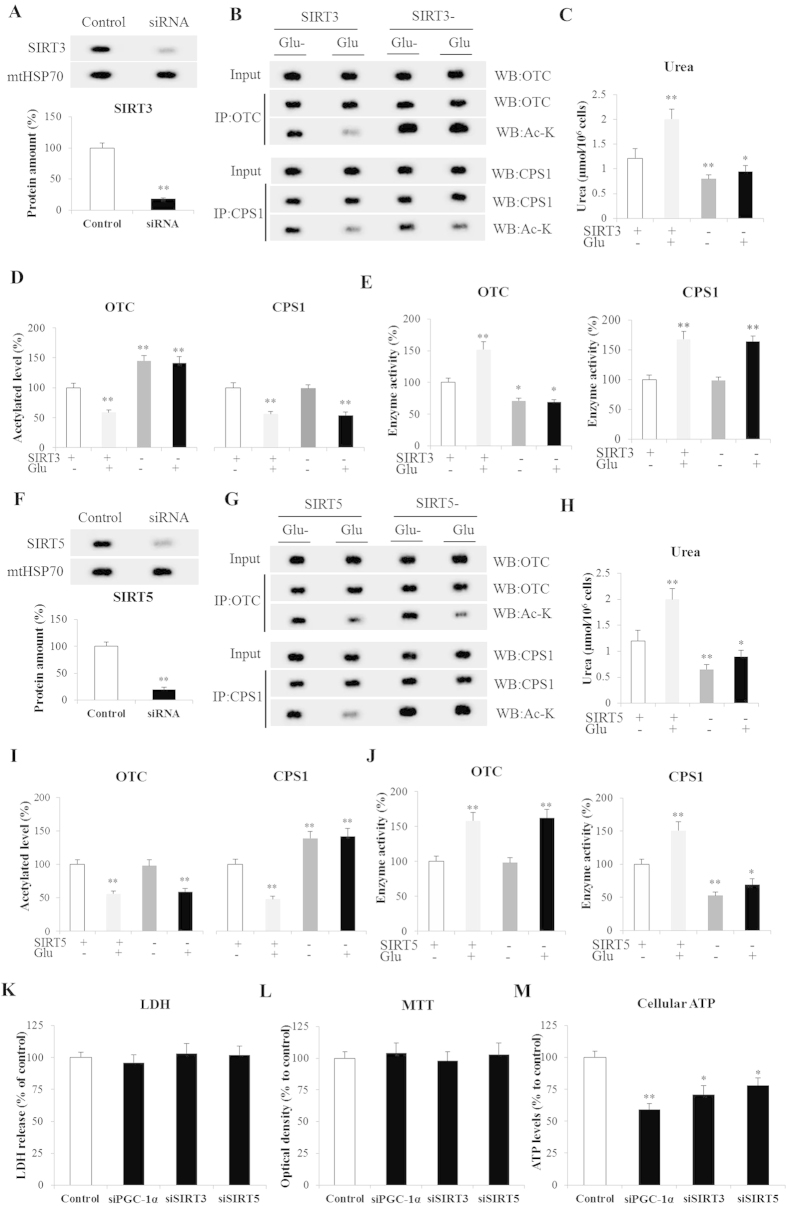
Effect of SIRT3 and SITR5 on ureagenesis in periportal hepatocytes. (**A**) SIRT3 expression in periportal hepatocytes; (**B**,**D**) Acetylated OTC and CPS1 in control and SIRT3 knockdown hepatocytes; (**C**) Urea in hepatocytes cultured media; (**E**) Activities of OTC and CPS1 in hepatocytes; (**F**) Expression of SIRT5 in periportal hepatocytes; (**G**,**I**) Acetylated OTC and CPS1 in control and SIRT5 knockdown hepatocytes; (**H**) Urea in hepatocytes cultured media. (**J**) Activities of OTC and CPS1 in hepatocytes; (**K**) Viability of gene knockdown hepatocytes as determined by leakage of cytosolic lactate dehydrogenase to the culture media; (**L**) Activity of mitochondrial succinate dehydrogenase (respiratory chain complex II), assayed by MTT; (**M**) Levels of hepatocyte ATP 24-hours afer transfection: SIRT3/5(+) Glu(−) were the control group. Glu(+) and Glu(−) represent with- and without glucagon in medium, respectively. SIRT3(−) and SIRT3/5(+) represent hepatocytes transfected with si SIRT3/5 and control siRNA, respectively. Data are expressed as means and standard errors of each different experiment (n = 4 for each group). *P < 0.05 from control group, **P < 0.01 from control group.

SIRT3 protein expression decreased approximately 82% after siRNA transfection (Fig. 6A). SIRT3 silencing strongly induced an increase in the level of acetylated OTC and a decrease of its activity (*P* < 0.01, Fig. 6B,D,E). In SIRT3-deficient hepatocytes there were no significant changes of acetylated CPS1 level and activity (*P* > 0.05, Fig. 6B,D,E), suggesting that SIRT3 efficiently deacetylated OTC, but not CPS1. Furthermore, ureagenesis was significantly decreased in hepatocytes with SIRT3 silencing both in the presence (*P* < 0.05) and absence of (*P* < 0.01) glucagon (Fig. 6C). Morever, SIRT5 was knockdown with SIRT5-specific siRNA and its protein expression was decreased approximately 80% in periportal hepatocytes (Fig. 6F). In SIRT5 knockdown hepatocytes, the acetylated CPS1 level was significantly higher (*P* < 0.01), while CPS1 activity was markedly lower both with (*P* < 0.05) and without glucagon treatments (*P* < 0.01) compared with control hepatocytes (Fig. 6G,I,J). However, there were no significant changes of acetylated OTC level and activity in lacking SIRT5 hepatocytes (*P* > 0.05, Fig. 6G,I,J). These results indicate that SIRT5 could upregulate CPS1 activity via deacetylation. For ureagenesis, lacking SIRT5 in periportal hepatocytes led to significantly decreased ureagenesis both in the presence (*P* < 0.05) and absence of (*P* < 0.01) glucagon (Fig. 6H). Morever, the viability of SIRT3, SIRT5 and PGC-1α knockdown hepatocytes assessed either by lactate dehydrogenase leakage or the activity of the mitochondrial complex II/succinate dehydrogenase (MTT assay) was unaffected (Fig. 6K,L).

## Discussion

The major finding in this study ceonters on the functional significance of PGC-1α, a transcriptional coactivator that promotes ureagenesis in fasting mice liver and periportal hepatocytes through mitochondria metabolic sensors SIRT3 and SIRT5. The presented evidences show that 1) PGC-1α null mice did not increase the output of ^15^N-labeled urea and ^13^C-labeled glucose in alanine perfused livers; 2) glucagon-induced ureagenesis from alanine is associated to up-regulation of PGC-1α in periportal hepatocytes; 3) PGC-1α could significantly induce expression of mitochondrial SIRT3 and SIRT5 in periportal hepatocytes under basal- and glucagon-stimulated conditions and 4) mitochondrial SIRT3 and SIRT5 could respectively deacetylate (activate) OTC and CPS1, thus promoting ureagenesis.

Ammonia detoxification via ureagenesis is critical for prevention of hyperammonemia and hepatic encephalopathy^38^. The urea cycle comes into play especially during fasting, when amino acids are catabolized for energy generating excess of ammonia, which must be detoxified^39^. It has been established that the regulation of the five enzymes in the urea cycle is complex: nutritional status upon fasting and glucagon have each been shown to elevate the activities of these enzymes in liver^39^,^40^. Glucagon as an important signaling molecule during fasting: it is vital to up-regulate hepatic ureagenesis on short- and long-term food deprivation and has been described to modulate hepatic urea cycle enzyme expression at multiple levels by transcriptional, posttranscriptional, and posttranslational mechanisms^40^,^41^,^42^,^43^. In the present study, an important role for glucagon in facilitating ureagenesis is suggested, and the mechanism of fasting-facilitated ammonia detoxication in hepatocyte was clarified.

In an *in vivo* experiment, this study was designed to investigate as broadly as possible the effects of fasting on the hepatic energy balance and metabolic homeostasis. Hepatic gluconeogenesis and ureagenesis were increased in fasted liver. In the cycle of feeding to fasting, the expressions and activities of key hepatic metabolic enzyme genes in gluconeogenesis (i.e., PEPCK, G6Pase) and ureagenesis (i.e., OTC, CPS1) were increased along with plasma glucagon concentration rised. This was so because AA maybe an important energy source during fasting and the flow of carbon from AA was promoted for hepatic gluconeogenesis, while most of the nitrogen entered urea cycle. The alterations in hepatic energy metabolism demonstrated that fasting produced an adaptive change in fuel usage and maintained metabolic homeostasis during the process from feeding to fasting^44^. Likewise, the expression of both transcriptional coactivator PGC-1α and mitochondrial metabolic sensors SIRT3 and SIRT5 were also increased in fasted mice liver in the present study. Their up-regulation may have an important functional implication in this process.

Using [^15^N,^13^C]alanine or ^15^N-ammonium chloride as metabolic tracers allowed identification of nitrogen sources for hepatic ureagenesis in wild-type mice perfused livers. Alanine is recognized as the most important amino acid donor for hepatic gluconeogenesis^45^ and a significant hepatic nitrogen donor for incorporation into urea. Interestingly, alanine plays an important role in the glucose-alanine cycle and, during starvation, taken up by the liver^45^. Our results indicate that alanine could significantly increase hepatic urea and glucose output in liver of wild-mice. In order to futher investigate the effect of PGC-1α on ureagenesis *in vivo*, PGC-1α null mice were obtained. We detected that almost no PGC-1α expression was in PGC-1α null mice liver. Notably, in PGC-1α null alanine-perfused livers there was no increase of urea and glucose production but it slightly decreased urea synthesis from ammonium chloride. This observation might suggest that the presence of a complex regulatory network in ureagenesis. These findings also revealed that alanine was an important source of carbon and nitrogen for hepatic gluconeogenesis and ureagenesis in response to fasting.

PGC-1α is an important transcriptional coactivator on fuel metabolism and a major regulator of cellular energy homeostasis. Hepatic PGC-1α is efficiently induced by food deprivation, promoting transcription of genes involved in gluconeogenesis and mitochondrial biogenesis and function^46^,^47^,^48^. Our experiments show that expression of PGC-1α was significantly increased in the presence of glucagon. This phenomenon in periportal hepatocytes was consistent with the *in vivo* experiments and also in agreement with previous findings that fasting could stimulate expression of gluconeogenesis through PGC-1α induced by glucagon^2^. In order to comprehensively understand its function, PGC-1α was knockdown and overexpressed in mice periportal hepatocytes, respectively. An unexpected discovery was that PGC-1α silencing decreased ureagenesis, while its overexpression significantly increased urea production. Moreover, the activities of CPS1 and OTC were high in PGC-1α overpression hepatocytes, while their activities were extremely low in PGC-1α knockdown hepatocytes. These results uncover that PGC-1α maybe an important urea cycle regulator in periportal hepatocytes in response to glucagon.

These interesting results attracted our attention, so the expression and acetylation levels of CPS1 and OTC were then detected. The expressions of CPS1 and OTC were unchanged in PGC-1α knockdown and overexpression hepatocytes, but their acetylation levels negatively correlated to their enzymatic activities. So it was reasonable to infer that PGC-1α promoted ureagenesis through modifying acetylation levels and enzymatic activity instead of enzymatic expression. Previous findings indicated that short-term regulation of urea synthesis primarily takes place at the level of substrate provision and enzyme activities, whereas long-term control is transcriptionally affected by changes in enzyme concentrations^10^.

It is remarkable that mitochondrial Sirtuins SIRT3 and SIRT5 as NAD-dependent protein deacetylases are reported as metabolic sensors that dynamically regulate cellular physiology and energy metabolism^3^. Interestingly, we found PGC-1α induced SIRT3 and SIRT5 expression, suggesting its involvement in hormone-modulated and PGC-1α-induced ureagenesis. This is in agreement with previous reports that PGC-1α and SIRTs are all cellular energy sensors, and their function and regulation are closely interrelated^15^. Taken together, PGC-1α could promote ureagenesis through mitochondria SIRT3 and SIRT5 in response to glucagon.

In addition, in this study the protein SIRT3 and SIRT5 expression was upregulated with glucagon study, suggesting its involvement in hormone-modulated and PGC-1α-induced ureagenesis. So SIRT3 and SIRT5 were individually knockdown in mice periportal primary hepatocytes, resulting in markedly decreased ureagenesis. Notably, OTC, but not CPS1 activity was decreased in SIRT3 knockdown hepatocytes. However, SIRT5 silencing only had a negative effect on CPS1 activity. These findings indicated SIRT3 and SIRT5 could deacetylate and activate OTC and CPS1, respectively. This was in agreement with the previous findings that SIRT3 played an important role in stimulating the urea cycle in response to dietary stress (fasting and CR)^16^ and starvation could trigger CPS1 activation by SIRT5-mediated deacetylation of the urea cycle enzyme^3^. These observations suggested that SIRT3 and SIRT5 play an important modulatory role in reprogramming mitochondria toward alternative fuel (i.e. AA) consumption during dietary stress.

In summary, during fasting PGC-1α promotes ureagenesis through deacetylating CPS1 and OTC by modulation of mitochondrial deacetylases SIRT3 and SIRT5 in mouse periportal hepatocytes. This mechanism may be relevant to ammonia detoxification and metabolic homeostasis in liver during fasting.

## Additional Information

**How to cite this article**: Li, L. *et al*. PGC-1α Promotes Ureagenesis in Mouse Periportal Hepatocytes through SIRT3 and SIRT5 in Response to Glucagon. *Sci. Rep.* **6**, 24156; doi: 10.1038/srep24156 (2016).

## Supplementary Material

Supplementary Information
