# Supplementary material for: PGC-1α Promotes Ureagenesis in Mouse Periportal Hepatocytes through SIRT3 and SIRT5 in Response to Glucagon
Source: Sci Rep. 2016 Apr 7;6:24156. doi: 10.1038/srep24156 (PMC4823758; doi:10.1038/srep24156)
Supplement: Supplementary Information [file srep24156-s1.pdf]

Supplementary information

PGC-1α Promotes Ureagenesis in Mouse Periportal Hepatocytes through SIRT3 and SIRT5 in Response to Glucagon

Lulu Li, Ping Zhang, Zhengxi Bao, Tongxin Wang, Shuang Liu & Feiruo Huang*

Department of Animal Nutrition and Feed Science, College of Animal Science and Technology, Huazhong Agricultural University, Wuhan 430070, China

* Corresponding author:

Feiruo Huang, Postal address: Huazhong Agricultural University, No.1 Shizishan Street, Hongshan District, Wuhan 430070, China, Phone: 86-27-87286912, Fax: 86-27-87280408, E-mail: huangfeiruo@mail.hzau.edu.cn

Supplementary Figures

Table S1. qPCR Primer

Gene	Forward, 5’-3’	Reverse, 5’-3’	Reference
OTC	TCCTGCTCAACAAGGCAGCTCTTA	TCACGGCCTTTCAGCTGTACTTGA	Walters et al. (2010)
CPS1	TGAGACAGGCCAAAGAGATTGGGT	TGCTCCTGGCCATTGTAGGTAACA	Walters et al. (2010)
Sirt3	GCTGCTTCTGCGGCTCTATAC	GAAGGACCTTCGACAGACCGT	Kong et al. (2010)
Sirt5	CTCCGGGCCGATTCATTTCC	GCGTTCGCAAAACACTTCCG	Buler et al. (2014)
PEPCK	CATATGCTGATCCTGGGCATAAC	CAAACTTCATCCAGGCAATGTC	Lin et al. (2004)
G6Pase	ACACCGACTACTACAGCAACAG	CCTCGAAAGATAGCAAGAGTAG	Lin et al. (2004)
PGC-1α	GCAGGTCGAACGAAACTGAC	CTCAGCCTGGGAACACGTTA	Buler et al. (2014)
18S	CGCCGCTAGAGGTGAAATTC	CCAGTCGGCATCGTTTATGG	Buler et al. (2014)

OTC, ornithine transcarbamoylase; CPS1, carbamoyl phosphate synthetase 1; Sirt3, sirtuin 3; Sirt5, sirtuin 5; PEPCK, phosphoenolpyruvate carboxykinase; G6Pase, glucose-6-phosphatase; PGC-1α: peroxisome proliferator-activated receptor gamma coactivator 1-alpha; 18S, 18S ribosomal RNA.

Supplemental References

1. Walters, M. W. & Wallace, K. B. Urea cycle gene expression is suppressed by PFOA treatment in rats. *Toxicol Lett* **197**, 46-50 (2010).
2. Kong, X. *et al.* Sirtuin 3, a new target of PGC-1alpha, plays an important role in the suppression of ROS and mitochondrial biogenesis. *PloS One* **5**, e11707 (2010).
3. Buler, M., Aatsinki, S. M., Izzi, V., Uusimaa, J. & Hakkola, J. SIRT5 is under the control of PGC-1α and AMPK and is involved in regulation of mitochondrial energy metabolism. *FASEB J* **28**, 3225-3237(2014).

4. Lin, J. *et al*. Defects in adaptive energy metabolism with CNS-linked hyperactivity in PGC-1α null mice. *Cell* **119**, 121-135 (2004).
